# Maximum Host Survival at Intermediate Parasite Infection Intensities

**DOI:** 10.1371/journal.pone.0002463

**Published:** 2008-06-18

**Authors:** Martin Stjernman, Lars Råberg, Jan-Åke Nilsson

**Affiliations:** Department of Animal Ecology, Lund University, Lund, Sweden; Oxford University, United Kingdom

## Abstract

**Background:**

Although parasitism has been acknowledged as an important selective force in the evolution of host life histories, studies of fitness effects of parasites in wild populations have yielded mixed results. One reason for this may be that most studies only test for a linear relationship between infection intensity and host fitness. If resistance to parasites is costly, however, fitness may be reduced both for hosts with low infection intensities (cost of resistance) and high infection intensities (cost of parasitism), such that individuals with intermediate infection intensities have highest fitness. Under this scenario one would expect a non-linear relationship between infection intensity and fitness.

**Methodology/Principal Findings:**

Using data from blue tits (*Cyanistes caeruleus*) in southern Sweden, we investigated the relationship between the intensity of infection of its blood parasite (*Haemoproteus majoris*) and host survival to the following winter. Presence and intensity of parasite infections were determined by microscopy and confirmed using PCR of a 480bp section of the *cytochrome-b*-gene. While a linear model suggested no relationship between parasite intensity and survival (*F* = 0.01, *p* = 0.94), a non-linear model showed a significant negative quadratic effect (quadratic parasite intensity: *F* = 4.65, *p* = 0.032; linear parasite intensity *F* = 4.47, *p* = 0.035). Visualization using the cubic spline technique showed maximum survival at intermediate parasite intensities.

**Conclusions/Significance:**

Our results indicate that failing to recognize the potential for a non-linear relationship between parasite infection intensity and host fitness may lead to the potentially erroneous conclusion that the parasite is harmless to its host. Here we show that high parasite intensities indeed reduced survival, but this effect was masked by reduced survival for birds heavily suppressing their parasite intensities. Reduced survival among hosts with low parasite intensities suggests costs of controlling parasite infections; however, the nature of such costs remains to be elucidated.

## Introduction

Parasitism is now acknowledged as an important selective force, comparable to predation and competition [1]. In host life history evolution, parasites have been proposed to play a significant role as mediators of fitness costs incurred as a result of reproductive effort or exaggeration of sexual ornaments [2], [3]. Empirical investigations of the importance of parasites in life history evolution have often used blood parasites of the family Plasmodiide (e.g. *Haemoproteus*, *Plasmodium* and *Leucocytozoon*) and their avian hosts as model systems. Results from laboratory studies on captive birds suggest that these parasites cause significant reductions in host fitness [4], [5]. However, results from studies of natural populations are mixed as both negative (e.g. [6]–[8]), and no effects of these parasites have been found (e.g. [9]–[11]). Also in other vertebrates the effect of parasites is often surprisingly weak [12]–[14].

The general approach in these studies has been to search for a linear relationship between infection status and fitness, implicitly assuming that infection intensities are only related to fitness through the negative effects (pathogenicity/virulence) of parasites (i.e. higher parasite intensities are always worse). However, it is often asserted that resistance to parasites is costly (e.g. [3], [15]–[17]). If so, highly resistant hosts, which maintain low infection intensities, could actually have lower fitness than less resistant hosts. Under this scenario, one could expect hosts with intermediate levels of infection intensity to have highest fitness, yielding a non-linear relationship between infection intensity and fitness. However, as far as we are aware, previous studies have not considered this possibility and therefore, any potential negative effects of parasites may have remained undetected. Here, we use a data set on infection intensities of blood parasites of the genus *Haemoproteus* in blue tits (*Cyanistes caeruleus*) to investigate the shape of the relationship between infection intensity and survival.

## Results

Of the 433 birds, 334 (77.1%) were found to be infected by *Haemoproteus* sp., as determined by microscopy. Of the 334 birds that were scored as positive by microscopy, we performed PCR analysis on 53 individuals. Sequencing showed that all of these were infected by *Haemoproteus majoris* strain PARUS1 [18]. Of the 99 birds that were scored as negative by microscopy, DNA samples were available for 93. PCR analysis confirmed that none of these were infected with *Haemoproteus sp.* Thus, we are confident that the birds that were excluded from the analysis of survival because they were uninfected by *Haemoproteus sp.* (see methods), were actually uninfected and did not just have very low levels of infection intensity, making them undetectable by microscopy. However, the PCR assay also showed that 55 of these birds were infected by *Plasmodium sp.* (38 with strain TURDUS1, 14 with BT7, and 3 with SGS1; [18]). As is often the case [19], these *Plasmodium* infections were of such low intensity that they were undetectable by microscopy. Because the sample sizes for birds infected with each of the *Plasmodium* strains are small, and because we do not have data on the intensity of these infections, we only performed survival analyses with birds infected by *Haemoproteus.*

Birds infected with *Haemoproteus* did not differ in survival from un-infected birds (average survival of non-infected birds: 30.3%, infected birds: 31.7%; n = 433, *χ* ^2^ = 1.06, *p* = 0.30, controlling for differences between sexes and years, *p* = 0.03 and *p*<0.0001, respectively).

**Figure 1:**
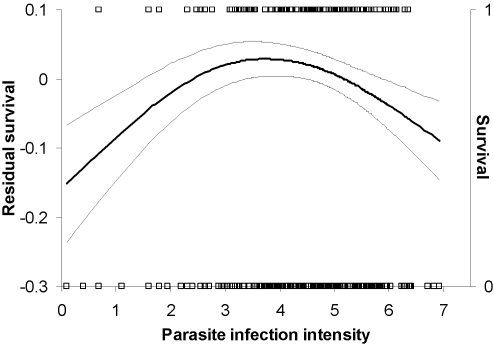
Survival of blue tits as a function of the infection intensity of *Haemoproteus majoris*. The lines show residual survival (cubic spline, thick solid line±SE, thin solid lines; left y-axis) after controlling for effects of sex (logistic regression; *χ* ^2^ = 5.74, *p* = 0.017), and year (*χ* ^2^ = 18.44, *p*<0.0001). Squares show individual data points (1 = survived; right y-axis).

A logistic regression considering only linear relationships did not show any relationship between parasite intensity and survival (estimate (SE) = 0.008 (0.106), *F* = 0.01, *p* = 0.94), controlling for significant effects of sex and year (sex: *F* = 4.65, *p* = 0.032; year: *F* = 7.54, *p = *0.0006). However, when including a quadratic effect of parasite intensity (non-linear model), we found a non-linear relationship between parasite intensity and survival (squared parasite intensity: estimate = −0.134 (0.067), *F* = 4.65, *p* = 0.032; parasite intensity: estimate = 1.04 (0.54), *F* = 4.47, *p* = 0.035; sex: *F* = 5.49, *p* = 0.020; year: *F* = 7.86, *p* = 0.0005). Neither age nor treatment or any two-way interactions were significant in any of the models. Visualization of the relationship with the cubic spline technique indicates that individuals with intermediate parasite intensities have highest survival probability (figure 1).

## Discussion

This study shows that a failure to acknowledge the possibility of a non-linear relationship between parasite infection intensity and host fitness might lead to the erroneous conclusion that the parasite under consideration is harmless to its host. While the linear effect of parasite intensity on survival was far from significant, our finding of a significant negative quadratic effect, combined with the presence of an intermediate optimum as revealed by cubic spline, suggests that high parasite intensities are indeed detrimental to the host. Moreover, our finding of reduced survival also for individuals maintaining low parasite intensities indicates that the costs of controlling the parasites at low levels may be strong enough to override the benefits. Interestingly, in a previous study in the same blue tit population we found highest survival for individuals with intermediate levels of humoral immune responsiveness [20]. Thus, these results stress the importance of considering trade-offs involving resistance when investigating fitness effects of parasite infections, especially in observational studies in natural populations.

We propose two possible explanations for the intermediate optimum for infection intensity. First, maintaining and/or using the immune system is assumed to be costly in terms of energy or immunopathology [15], [17], [21]. Improved resistance against parasites through enhanced immune function reduces the cost incurred from the parasite infection while increasing the cost of the immune system, yielding a pattern of maximum fitness for intermediate intensities. Second, as different components of the immune system are effective against different kinds of parasites, with a negative feedback system between these components, resistance against one particular parasite might be negatively related to resistance against another [22]. Individuals mounting a strong response against one parasite might expose themselves to other parasites with negative net effects for fitness, again yielding highest fitness for individuals with intermediate intensities of any specific parasite type.

To conclude, by considering a non-linear relationship between parasite intensity and survival, we were able to show that parasites can exert a selective pressure on its host as high parasite intensities resulted in reduced survival. Whether this was a direct effect of *Haemoproteus* infections, or a result of positively correlated infections from other parasites, remains to be shown. Still, it shows that parasites may be an important selective force in this population of blue tits, selecting for improved parasite resistance. However, as we also found that strong resistance (as indicated by low parasite intensities) reduced survival, suggesting that resistance carries costs, intermediate rather than maximal levels of resistance are favoured.

## Materials and Methods

This study is based on data from 2000–2002, including 433 adult individuals from a nest box breeding blue tit population at Revingehed, approximately 20 km east of Lund, southern Sweden. Breeding birds were caught during nestling feeding 14 days after hatching of their young and age (second calendar year bird, 2Y, or older) and sex determined according to Svensson [23].

Infection status of all birds was determined by microscopy. A blood sample was taken from the jugular vein and a small drop was used to make a blood smear. Smears were fixed in absolute methanol (3 minutes) and later stained with modified Giemsa stain (ACCUSTAIN®, SIGMA Diagnostics, diluted 1∶20 in ddH_2_O). Parasite status (prevalence and intensity) was determined by light microscopy under 1000× magnification (oil immersed), counting the number of parasites in 10^4^ red blood cells. Only *Haemoproteus* sp. was found in adequate numbers such that intensity could be accurately determined and from hereon we therefore only consider this parasite (other parasite genera occur at low prevalence and with average intensities well below 1 per 10^4^ red blood cells; see [24] for further details). Repeatability of parasite intensity counts using this method is high, both within and between smears from the same sampling occasion (R = 0.97–0.99; [24]) as well as between samples taken approximately 20 days apart (R = 0.70–0.75; M Stjernman, L, Råberg and J-Å Nilsson unpublished ms). Hence, our parasite intensity estimates should reflect the intensity level maintained by individual birds. Parasite intensities were log-transformed (ln(parasite intensity + 1)) to meet requirements of parametric tests.

A subset of the birds were also subject to PCR-based analysis to 1) verify that individuals scored as uninfected by microscopy were actually uninfected and not just had very low infection intensities, and 2) determine which parasite strains occurred in our blue tit population. Briefly, 20 μl blood was collected in SET-buffer. DNA was extracted by the proteinase K/phenol-chloroform method, following standard procedures [25]. 480 bp of the *Haemoproteus* and *Plasmodium cytochrome b* gene was amplified with a nested PCR, as described in Hellgren et al. [18]. This method has been shown to consistently detect infection intensities down to 1 parasite per 1×10^5^ red blood cells and at least 50% of intensities at a concentration of 1 parasite per 1×10^6^ red blood cells [18]. Positive samples were sequenced to determine parasite species and strain following methods described in Hellgren et al.[18].

Survival was based on whether or not birds were recaptured during the following winter (during nightly nest box visits in January–March) or the following breeding season. A bird was considered to have survived if it was alive at least in the winter following the focal sampling occasion.

Non-infected birds could either be resistant to infection by this parasite or they might not have been exposed to infectious vectors. Data from birds sampled for blood parasites more than once in successive years show that very few (1 in 116; i.e, <1%) loose their *Haemoproteus* infection (M. Stjernman, J-Å Nilsson, L. Råberg unpublished ms), indicating that the vast majority of the birds scored as non-infected have never been exposed to the parasite. Therefore, to ensure that all birds had been exposed to the parasite, we only included infected birds in the analysis of survival.

The relationship between parasite intensity and survival were analysed with logistic regression using PROC GENMOD in SAS 9.1 with binomial error distribution, a logit link function, type 3 analysis and dscale option to control for overdispersion [26]. Linear and non-linear relationships between parasite intensity and survival was analysed by including parasite intensity and squared parasite intensity as continuous variables in the models. As data were collected over three years and includes birds of both sexes and two age classes (2Y or older), all models also initially contained year, sex and age. Further, during the course of this study, breeding birds were subject to brood size manipulations. Two days after hatching of the young, brood size was increased (by 1/3), decreased (by 1/3) or left unchanged (controls). Since this treatment might affect both parasite intensities and survival we also included treatment in the models [24]. In the initial models we also included all two-way interactions. The large number of parameters in the model limited our ability to test for higher order interactions since the sample size for such interactions was very small. Final models were reached by excluding effects with *p*>0.10 in a backwards stepwise manner.

We tested two different models: one excluding (linear model) and one including (non-linear model) squared parasite intensity. To verify the existence of an intermediate optimum, as indicated by a significant quadratic effect in the non-linear model, we visualized the relationship between parasite intensity and survival with the cubic spline technique [27].
